# Interannual Recruitment Dynamics for Resident and Transient Marsh Species: Evidence for a Lack of Impact by the Macondo Oil Spill

**DOI:** 10.1371/journal.pone.0058376

**Published:** 2013-03-13

**Authors:** Ryan M. Moody, Just Cebrian, Kenneth L. Heck

**Affiliations:** 1 Dauphin Island Sea Lab, Dauphin Island, Alabama, United States of America; 2 Department of Marine Sciences, University of South Alabama, Mobile, Alabama, United States of America; Institute of Marine Research, Norway

## Abstract

The emulsification of oil at the Deepwater Horizon (DWH) well head relegated a large proportion of resultant hydrocarbon plumes to the deep sea, facilitated the incorporation of oil droplets into microbial and planktonic food web, and limited the severity of direct, wetland oiling to coastal Louisiana. Nevertheless, many transient fish and invertebrate species rely on offshore surface waters for egg and larval transport before settling in coastal habitats, thereby potentially impacting the recruitment of transient species to coastal nursery habitats quite distant from the well site. We compared the utilization of salt-marsh habitats by transient and resident nekton before and after the DWH accident using data obtained from an oyster reef restoration project in coastal Alabama. Our sampling activities began in the summer preceding the DWH spill and continued almost two years following the accident. Overall, we did not find significant differences in the recruitment of marsh-associated resident and transient nekton in coastal Alabama following the DWH accident. Our results, therefore, provide little evidence for severe acute or persistent oil-induced impacts on organisms that complete their life cycle within the estuary and those that spent portions of their life history in potentially contaminated offshore surface waters prior to their recruitment to nearshore habitats. Our negative findings are consistent with other assessments of nekton in coastal vegetated habitats and bolster the notion that, despite the presence of localized hydrocarbon enrichments in coastal habitats outside of Louisiana the most severe oil impacts were relegated to coastal Louisiana and the deep sea. Analyzing all the information learned from this accident will undoubtedly provide a synthesis of what has or has not been affected in the Northern Gulf of Mexico, which when put in context with oil spill studies elsewhere should improve our ability to avert and manage the negative consequences of such accidents.

## Introduction

The Deepwater Horizon oil flow began on April 20, 2010 and released an estimated 4.9 million barrels of crude oil into the Gulf of Mexico over a period of 84 days [1], [2]. In contrast to the Exxon Valdez spill, which directly oiled nearby coastal habitats [3], [4], oil from Deepwater Horizon (DWH) was emulsified at the well head, producing large, sub-surface hydrocarbon plumes in addition to surface slicks [2]. Oil droplets produced by dispersants and natural biodegradation resulted in the incorporation of hydrocarbons into microbial and planktonic food webs, which constitute the prey base for many organisms and their planktonic larvae [5]. The deleterious effects of dispersed oil from this accident on the survival, growth and recruitment of organisms to coastal habitats in the Gulf of Mexico are largely unknown.

Coastal impacts from the DWH spill were most severe in the marshes of coastal Louisiana, resulting in the loss of fringing marsh grasses and enhanced shoreline erosion [6], [7]. Much of the weathered material that approached the coasts of Mississippi, Alabama and Florida was intercepted by their barrier islands and only light to moderate oiling was reported in these states’ coastal marshes since the accident [8]. Salt marshes provide critical nursery habitat for the juvenile stages of many transient fish and invertebrate species (i.e., species known to utilize separate juvenile and adult habitats [9]), which rely on offshore surface waters for egg and larval transport before settling in coastal vegetated habitats. Coastal marshes in the northeastern Gulf were subjected to acute, light oiling particularly in June and July 2010 [10]. In contrast, early ontogenetic stages of transient species were subjected to significantly longer periods of oil exposure in offshore surface waters [8], [10].

Spring is a key spawning and recruitment period for many fishes and invertebrates, including commercially important penaeid shrimp and many finfish species, which can spend weeks in offshore surface waters as larvae [11], [12]. The blue crab (*Callinectes sapidus*) also exhibits a spring recruitment period, although peak recruitment occurs in the summer and fall [13]. Oil exposure in offshore surface waters can smother eggs and larvae, and foul feeding and respiratory structures in fish and shellfish. Furthermore, polycyclic aromatic hydrocarbons (PAHs), which are found in both fresh and weathered oil, can generate genetic abnormalities in fish eggs and larvae [14], [15]. Thus, the DWH accident may have produced, either directly or indirectly, detrimental impacts on eggs and larvae offshore, and led to reduced recruitment rates of juveniles to coastal nursery habitats quite distant from the well site.

An oyster-reef restoration project conducted from August 2009 to February 2012 in coastal Alabama provided a unique opportunity to assess the potential impacts on marsh-associated nekton assemblages before and after the DWH spill. Alabama beaches received weathered crude oil in June 2010, primarily in the form of sheens and tar balls [16], and coastal wetlands received light oiling in the form of highly weathered tar balls; [8]. Elevated hydrocarbons at the restoration site were detected in marsh sediments in June and July 2010, followed by a decline in hydrocarbons in September 2010 as a result of microbial degradation [17].

Given the large offshore area affected by oil and dispersants, and the relatively light oiling experienced by salt marshes in coastal Alabama, we predicted that oil-impacts would produce a larger decline in marsh-associated juveniles that rely on offshore habitats prior to their recruitment to estuarine habitats (“transient” marsh species; e.g. blue crabs, *Callinectes sapidus* and penaeid shrimp) relative to estuarine species that complete their life cycles in coastal waters (“resident” species; e.g., fundulid fishes and palaemonetid shrimp; [9]). Alternatively, we predicted system-wide impacts to both offshore and inshore habitats would be conducive to concomitant and similar declines in both groups. Lastly, we predicted that the presence of reefs would both facilitate nekton recruitment and potentially shield the shoreline from oil.

**Figure 1:**
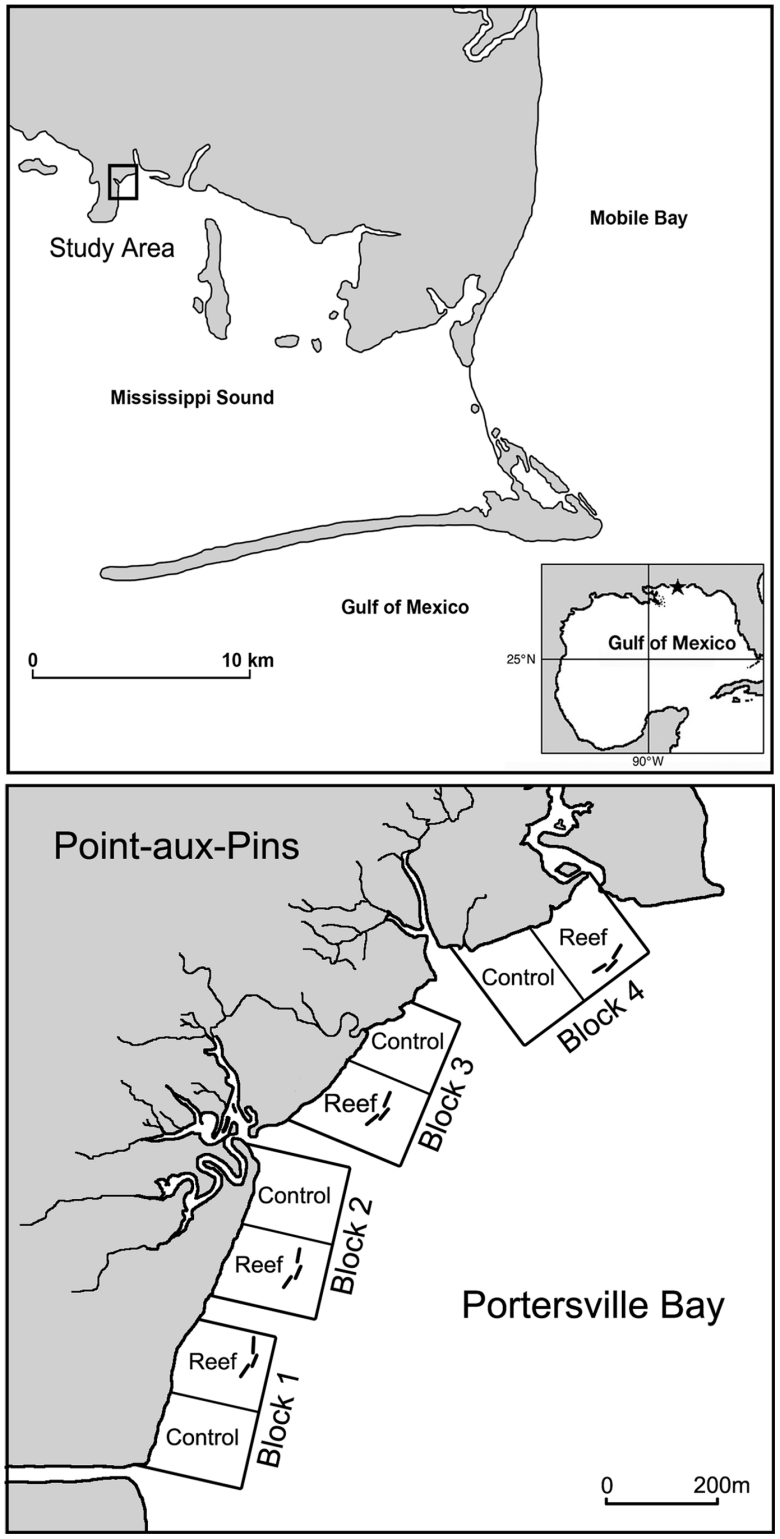
Location of study site within Mississippi Sound, Alabama. The study site consisted of four experimental blocks, each containing one reef and one control plot. Oyster reefs were located 111 m from shore and were composed of three, 25-m long modules (total diameter = 65 m). Reef and control plots were separated by 75 m, and blocks were separated by a minimum of 100 m.

To test these predictions, we examined divergences in the temporal trajectories of the abundances of transient and resident species obtained with tidal fyke nets designed to sample organisms within fringing marsh vegetation. “Transient” and “resident” designations were assigned based on life-history accounts from the literature. Our dataset consists of 144 fyke net catches collected over 18 dates within fringing salt-marsh habitat in coastal Alabama (Figure 1). Four oyster reefs, each paired with an adjacent control plot, were constructed to test for differences in marsh-associated nekton abundances among wave-sheltered (reef) and exposed (control) plots. Our analyses include comparisons of nekton abundances and biomass among years (one pre-spill year [2009] and two post-spill years [2010 and 2011]) and reef treatments for individual, numerically dominant species, and for community assemblages.

## Results

We collected 41,510 individual organisms representing more than 42 taxa in coastal Alabama between the summer of 2009 and winter of 2012. The resident daggerblade grass shrimp, *Palaemonetes pugio*, overwhelmingly dominated catches in terms of abundance, comprising 84.9% of the overall catch and 97.55% of resident organisms (Table S1 in File S1). When *P. pugio* was removed from the data set, resident organisms constituted 14.19% of the remaining catch and transient organisms 85.8%. Commercially important blue crab, *Callinectes sapidus*, and penaeid shrimp (*Farfantepenaeus aztecus* and *Litopenaeus setiferus*) accounted for 45.15% and 36.04% of the total catch, respectively, after removing *P. pugio*. All blue crabs and penaeid shrimp were juveniles or subadults, and all transient fishes fell within juvenile size classes.

**Figure 2:**
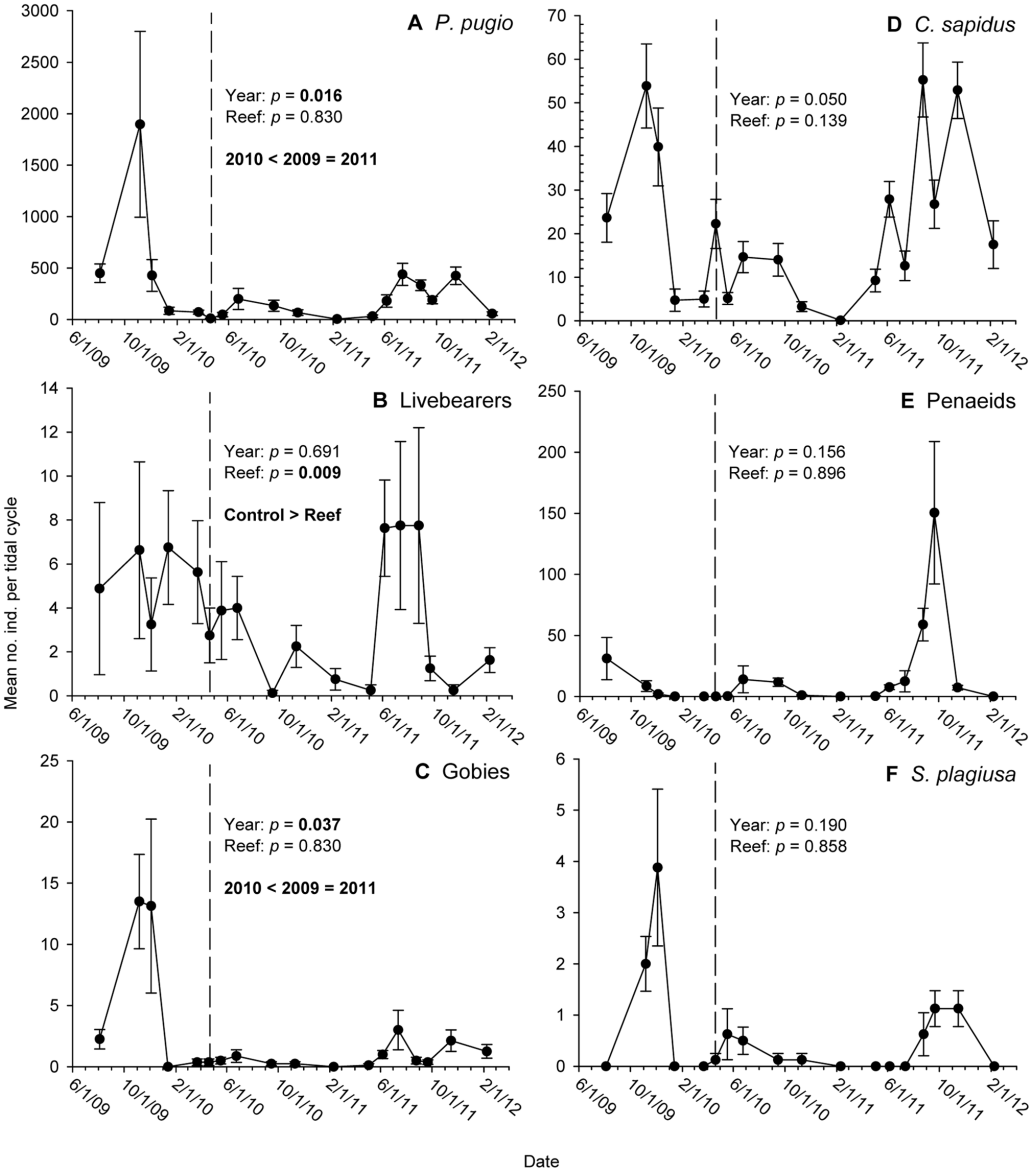
Time-series abundance plots for numerically dominant resident and transient species, 2009–2011. Mean abundances of (A) *Palaemonetes pugio*; (B) livebearers; (C) gobies; (D) *Callinectes sapidus*; (E) penaeid shrimp; and (F) *Symphurus plagiusa*. Means are derived from eight traps per sample date. Error bars: +1 SE.

Abundances of *P. pugio* differed among years (F_1,120_ = 5.57; *p* = 0.016) but not between reef treatments (F_1,120_ = 0.05; *p* = 0.830; Table S2A in File S1, Figure 2A). *P. pugio* abundances were highest in the pre-spill year (2009), declined in the first post-spill year (2010), and returned to pre-spill levels in the second post-spill year (2011). Livebearer (species in the families fundulidae, poeciliidae and cyprinodontidae) abundances did not differ among years (F_1,120_ = 0.38; *p* = 0.691) but did differ among reef treatments (F_1,120_ = 6.89 *p* = 0.010; Table S2B in File S1, Figure 2B). Abundances of livebearers were highest in control plots (mean abundance ±1 SE: 5.36±1.04) and lowest in reef plots (2.13±0.35). Abundances of gobies differed among years (F_1,120_ = 4.13; *p* = 0.037), exhibiting a post-spill decline in 2010 followed by a return to pre-spill abundances in 2011. Goby abundance did not differ among reef treatments (F_1,120_ = 0.17; *p* = 0.682; Table S2C in File S1; Figure 2C). Individual analyses of the three most abundant transient taxa yielded no significant differences in abundance among years (*p*-value range: 0.050–0.190) or between reef treatments (*p*-value range: 0.139–0.896; in File S1, Table S3A–C Figure 2D–F).

Whenever we did not find significant differences among the yearly means, we tested for differences in peak annual recruitment by comparing sample dates with the highest mean abundances among years. Annual maxima for livebearers and blackcheek tonguefish (*S. plagiusa*) did not differ among years (livebearers: *p = *0.885; *S. plagiusa*: *p = *0.060), but significant differences were detected for *C. sapidus* and penaeids (*p* = 0.012 and *p = *0.005, respectively). Annual maxima of *C. sapidus* did not differ between 2009 and 2011, but maxima in both years were higher than in 2010. Penaeid annual maxima were higher in 2011 than in 2009 and 2010, with the annual maxima in these two latter years not differing significantly from each other.

**Figure 3:**
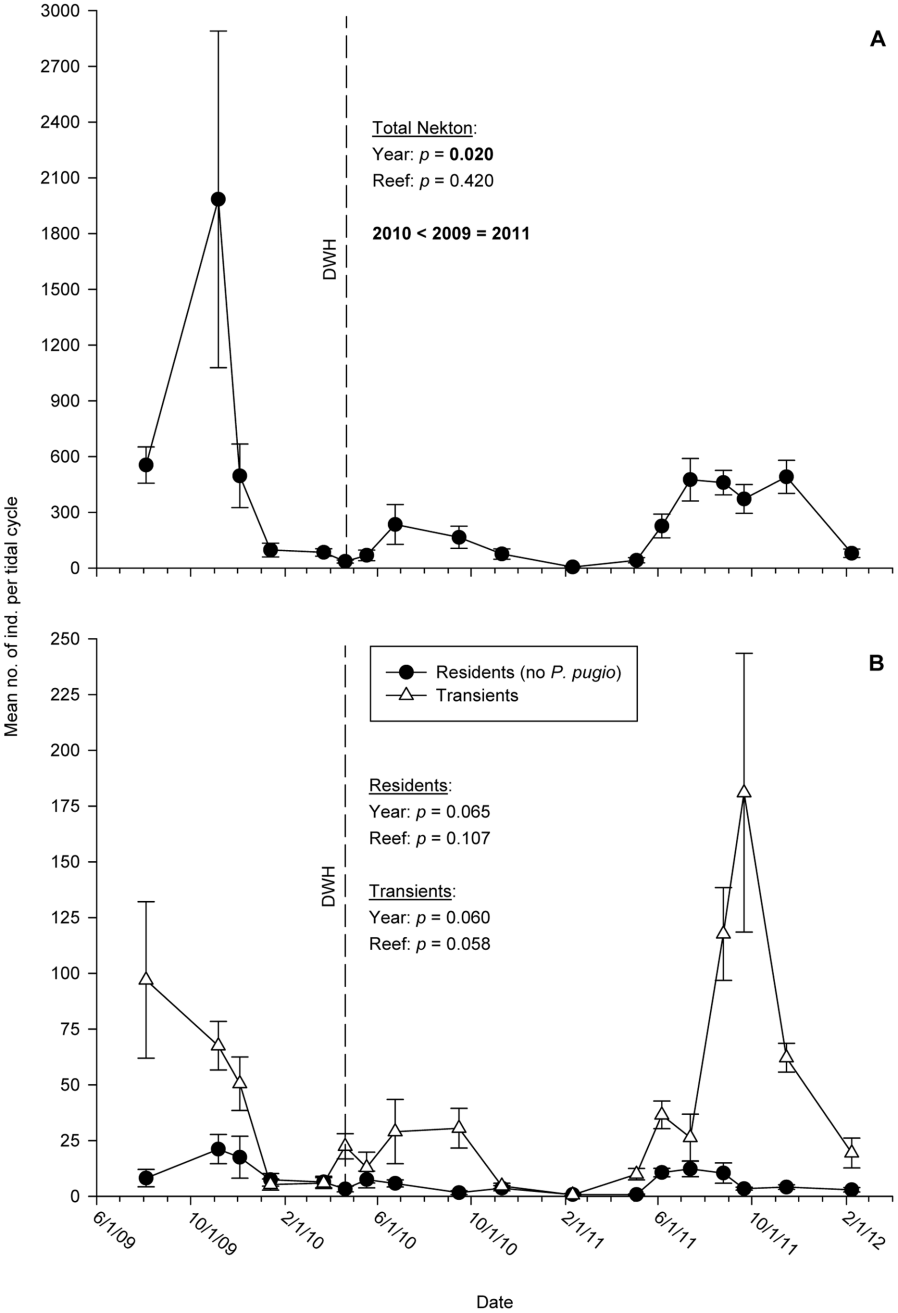
Time-series plots of mean nekton abundances, 2009–2011. Mean abundances of (A) total nekton catches and (B) transient and resident species. Abundances of grass shrimp (*Palaemonetes pugio*) have been excluded from resident means in (B). Means are derived from eight traps per sample date. Error bars: +1 SE.

Total nekton abundance differed significantly among years (F_2,120_ = 5.12; *p = *0.020) but not between reef treatments (F_1,120_ = 0.66; *p = *0.420; Table S4A in File S1; Figure 3A). As expected, among-year differences in total abundance closely tracked those of the numerically dominant *P. pugio*. Abundances of resident organisms with *P. pugio* removed from the data set did not differ among years (F_2,120_ = 3.29; *p* = 0.065) or between reef treatments (F_1,120_ = 2.63; *p = *0.107; Table S4B in File S1; Figure 3B). Similarly, the total abundance of transient organisms did not differ among years (F_2,120_ = 3.42; *p = *0.060) or reef treatments (F_1,120_ = 3.67; *p = *0.058; Table S4C in File S1; Figure 3B). Annual maxima of resident nekton did not differ among years (*p = *0.090), but significant variability was detected for transient taxa (*p* = 0.013). Namely, transient maximum abundance was higher in 2011 than in 2010 although no other significant differences were found between years.

**Figure 4:**
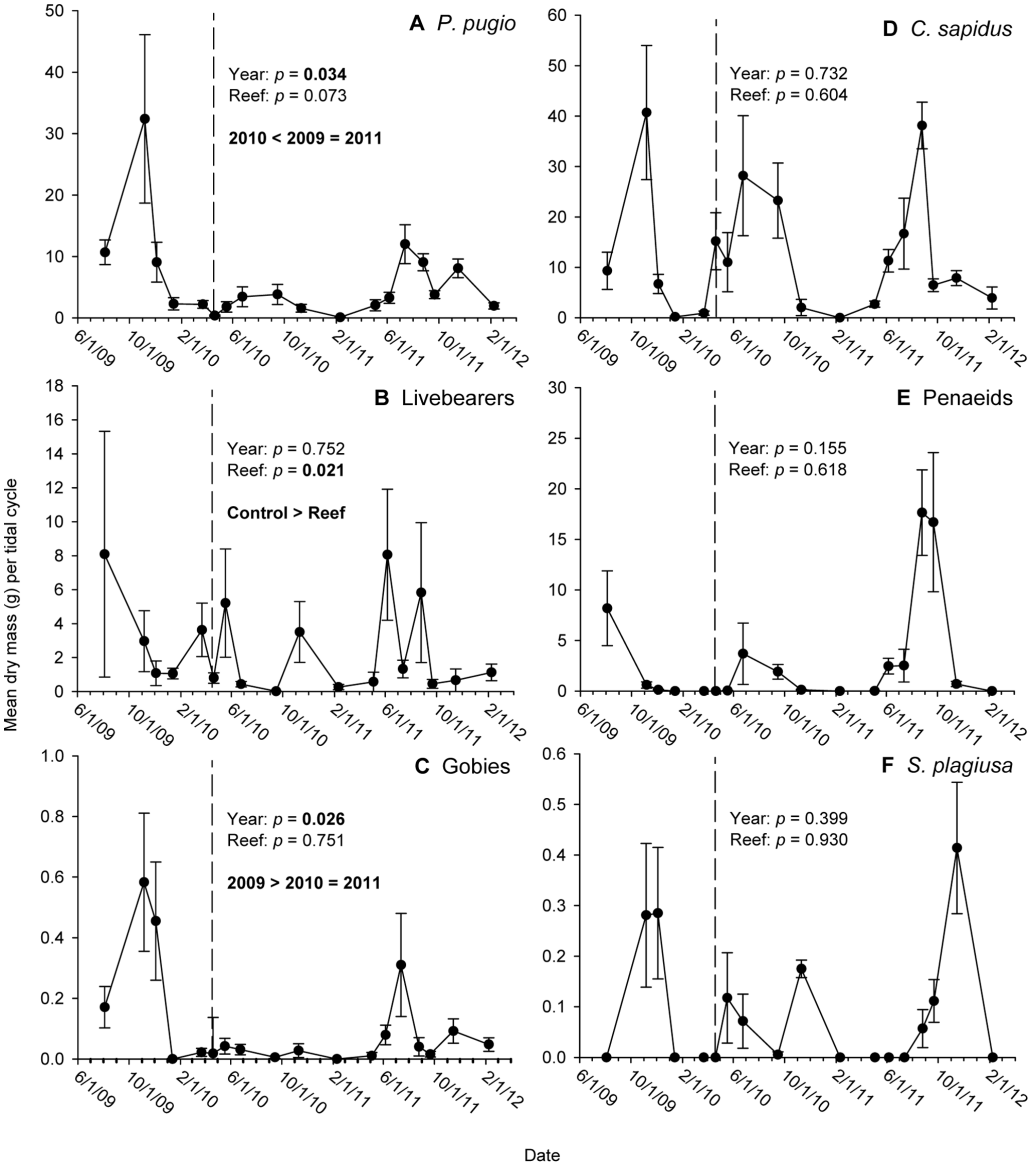
Time-series biomass plots for numerically dominant resident and transient species, 2009–2011. Mean biomass of (A) *Palaemonetes pugio*; (B) livebearers; (C) gobies; (D) *Callinectes sapidus*; (E) penaeid shrimp; and (F) *Symphurus plagiusa*. Means are derived from eight traps per sample date. Error bars: +1 SE.

Significant among-year variability in biomass was detected for the residents *P. pugio* (F_2,120_ = 4.26; *p = *0.034) and gobies (F_2,120_ = 4.71; *p = *0.026), but not livebearers (F_2,120_ = 0.29; *p = *0.752; Table S5 in File S1; Figure 4A–C). Among-year differences in *P. pugio* tracked trends in mean abundance, but goby biomass did not reflect the 2011 recovery in abundance. No among-year differences in biomass were detected for the three numerically dominant transient taxa (*p-*value range: 0.155–0.732; in File S1, Table S6A–C, Figure 4D–F). With the exception of livebearer biomass, which was higher in control than in reef plots, we did not detect differences in biomass among reef treatments for any of the remaining five taxa (*p*-value range: 0.094–0.930). Interannual biomass maxima did not differ significantly among years for livebearers, *C. sapidus*, penaeids or *S. plagiusa* (*p*-value range: 0.053–0.911).

**Figure 5:**
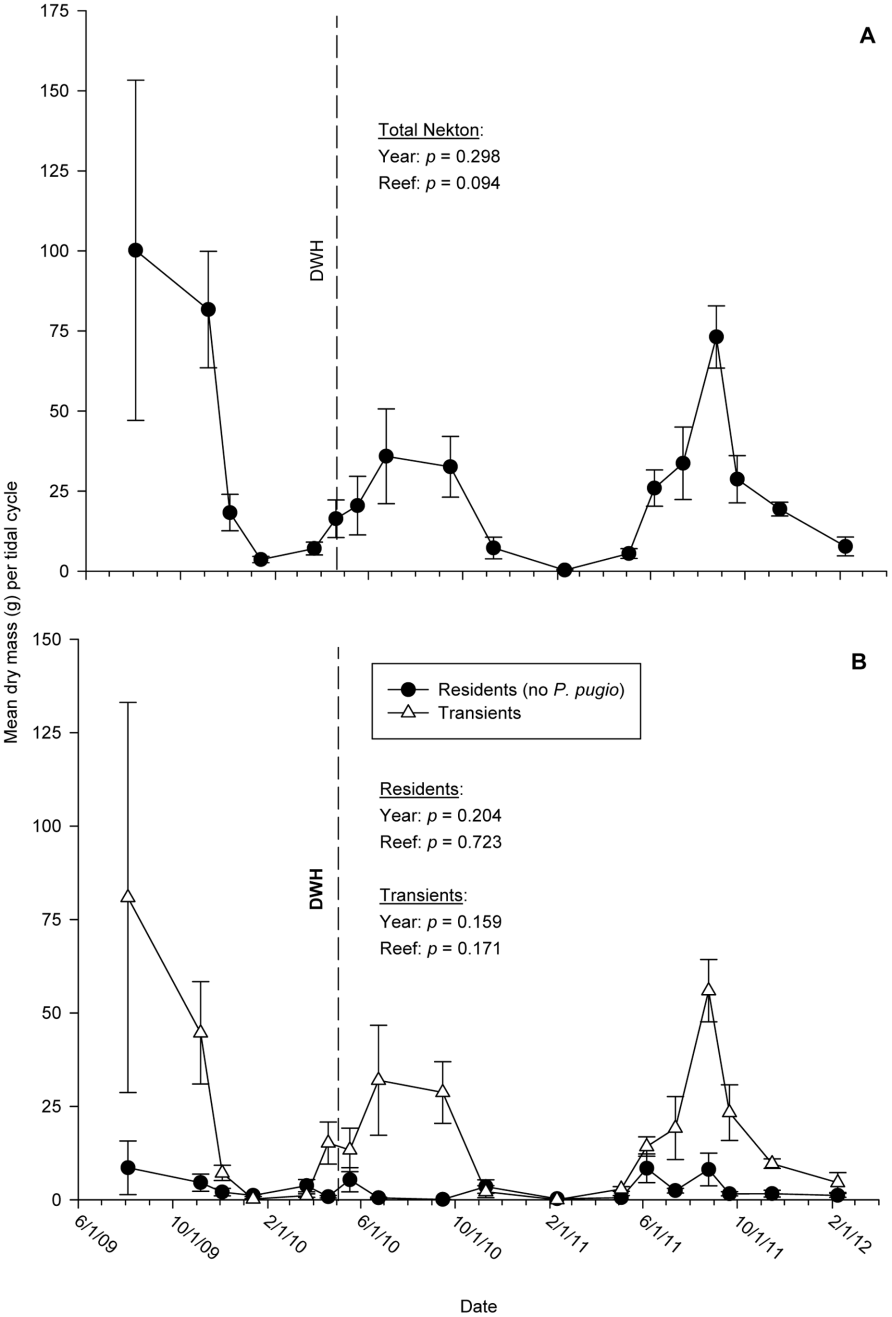
Time-series plot of mean nekton biomass, 2009–2011. Mean biomass of (A) total nekton catches, and (B) transient and resident species. Biomass of grass shrimp (*Palaemonetes pugio*) has been excluded from resident means in (B). No significant differences in mean biomass were detected among years for any group (Table S2 in File S1). Means are derived from eight traps per sample date. Error bars: +1 SE.

Total nekton biomass did not differ significantly among years (F_2,120_ = 1.31; *p = *0.298) or between reef treatments (F_1,120_ = 2.85; *p = *0.094; in File S1, Table S7A, Figure 5A). Mean resident (without P. pugio) and transient biomass did not differ among years (residents: F_1,120_ = 1.77, *p = *0.204, Table S7B in File S1, Figure 5B; transients: F_1,120_ = 2.09; *p = *0.159, Table S5C in File S1, Figure 5B) or between reef treatments (residents: F_1,120_ = 0.13; *p = *0.723; Table S7B in File S1, Figure 5B; transients: F_1,120_ = 1.90; *p = *0.171, Table S5C in File S1; Figure 5B). Interannual maxima did not differ significantly for any of the three groups (*p*-value range: 0.060–0.565).

**Figure 6:**
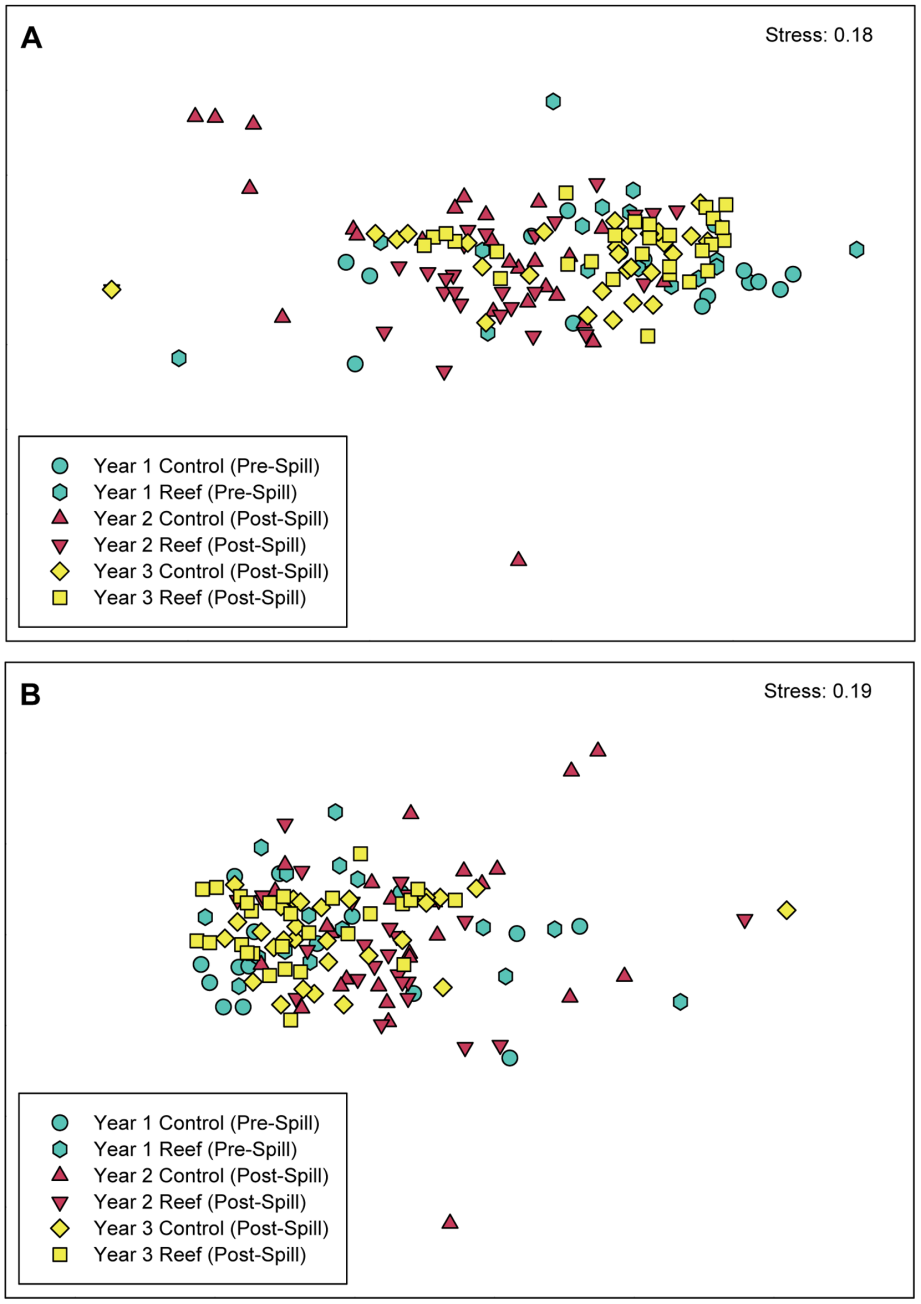
Community composition of marsh-associated fishes and invertebrates, 2009–2011. Multi-dimensional scaling plots for (A) total nekton biomass and (B) total nekton abundance compared among years and reef treatments (colored symbols). Each datum represents a single fyke-net sample.

Community structure in terms of abundance or biomass as analyzed using multidimensional scaling (MDS) ordination did not change among years (Global *R = *0.163 and *p* = 0.001 for abundance; Global *R* = 0.147 and *p = *0.001) for biomass) and between reef treatments (Global *R* = −0.009 and *p = *0.732 for abundance; Global *R* = −0.01 and *p = *0.766 for biomass; Figures 6A and B).

## Discussion

Although abundance and biomass of two resident groups, *P. pugio* and gobies, declined significantly following the DWH accident, abundances of both groups, and biomass of *P. pugio*, returned to pre-spill abundances by 2011. Goby biomass was the only metric that remained suppressed in 2011, however. Due to the numerical dominance of *P. pugio*, total nekton abundance also exhibited a significant post-spill decline, followed by a 2011 recovery to pre-spill levels. When *P. pugio* was removed, however, among-year differences in resident abundances disappeared. We did not observe a significant decline in the abundance or biomass of transients, the faunal component most likely to have experienced offshore oil impacts, either among individual species or as a community. Some variation was detected among transient abundance maxima, but differences were either followed by a 2011 recovery or were not related to the timing of the spill. Overall, we did not find significant divergences in the trajectories of marsh-associated resident and transient nekton in coastal Alabama following the DWH accident. Depsite widespread contamination of offshore waters and, to a lesser extent, coastal waters,ur results provide little evidence for large-scale acute or persistent oil-induced impacts on organisms that complete their life cycle within the estuary and those that spent portions of their life history in offshore surface waters prior to their recruitment to nearshore habitats. Our findings are consistent with other published assessments of nekton in coastal vegetated habitats [18] and bolster the notion that, despite the presence of localized hydrocarbon enrichments in coastal habitats outside of Louisiana [16], the most severe oil impacts were largely relegated to the deep sea [2], [19].

Significant, post-spill declines were detected in total nekton abundance, but this was attributed to the numerically dominant *P. pugio. Palaemonetes pugio* abundances were temporally variable and consistently 2–3 orders of magnitude higher than that of any other taxa and were responsible for driving significant among-year differences in total nekton abundance. When *P. pugio* was disregarded, however, among-year differences in resident communities were no longer present. This illustrates the driving effect of *P. pugio* on total nekton abundance, and suggests that the stability of livebearers, which accounted for 52.6% of the remaining residents, superseded among-year differences in goby abundance, which accounted for 32.3% of residents after the removal of *P. pugio*. In contrast to resident taxa, total transient abundance did not differ among years, which is not surprising considering that *C. sapidus* and penaeid shrimp, neither of which exhibited post-spill declines, comprised 45.15% and 36.04% of transients, respectively.

Statistical trends in the biomass of individual resident and transient species were similar to those found for abundance, but the transients *C. sapidus* and penaeid shrimp were the predominant drivers due to the relatively small contribution of *P. pugio* to total biomass. Biomass did not differ among years for *C. sapidus* or penaeid shrimp, which resulted in a lack of among-year differences in total nekton biomass and total transient biomass. Total resident biomass did not differ among years due to the stabilizing effects of livebearers, as was the case for total resident abundance. In summary, nekton biomass reflected patterns of abundance for individual species, but total community biomass assessments fluctuated to a lesser degree than abundance through time. In accordance with our findings for individual faunal groupings, our multivariate analyses of community structure did not distinguish among-year differences for abundance or biomass.

Annual maxima differed significantly among years for abundances of both *C. sapidus* and penaeid shrimp, but each followed different trajectories. Blue crabs experienced a significant post-spill decline in 2010, but recovered to pre-spill abundances in 2011. In contrast, penaeid abundances in the first post-spill year did not differ from pre-spill levels, and both periods were lower than 2011 catches. It is reasonable to deduce a possible subtle, yet acute, impact on peak *C. sapidus* abundances followed by a rapid recovery in the subsequent post-spill year, but this was not the case for penaeid shrimp. Overall, pre-spill maxima for total transient abundances did not decline relative to the pre-spill period.

Due to the light and variable nature of oiling within our study site and surrounding shorelines, it is possible that mobile organisms within the study area were able to avoid oiled shorelines. Oil was, however, detected within the sediment of coastal marshes in September 2010 [17] following a short-term window of exposure for coastal Mississippi, Alabama and Florida from June to July 2010 [10]. This period of exposure potentially played a role in the post-spill declines of *P. pugio* and goby abundance and biomass in 2010. As benthic feeders, both groups would have been the most susceptible to sediment contamination, yet *P. pugio* and gobies exhibited a recovery to pre-spill abundances in 2011. Previous research suggests that salt-marsh nekton and infauna are relatively robust to short-term, direct impacts of heavily weathered oil [20], [21]. Goby biomass was the only metric in our study that remained suppressed in 2011, however, making it difficult to distinguish between an acute impact of marsh oiling on 2010 abundances of *P. pugio* and gobies, or a simple reflection of interannual variability in recruitment due to other factors.

In contrast, given the widespread nature of oiling in offshore surface waters, it is unlikely that the larvae and juveniles of transients escaped exposure prior to recruiting to coastal vegetated habitats. While it is possible that small pools of larvae inhabiting patchy, unaffected surface waters were able to enter coastal habitats with minimal exposure, it is likely that the vast majority of young transient species experienced significant exposure to oil at some point during their early ontogeny. Nevertheless, it is clear from our data that nekton recruitment was sufficient to maintain pre-spill abundances and biomass of transient species despite such exposure or loss. Whether larvae were able to actively avoid contaminated surface waters en masse, a highly unlikely proposition [10], our results suggest that young transient organisms are suited to cope with large-scale perturbations in the northern Gulf of Mexico.

The DWH accident coincided with the end of a particularly severe winter in the Northern Gulf of Mexico, which affected marine mammals and many other marine organisms [22]. This and other factors driving interannual variability unrelated to the spill provide additional explanations for the variability in *P. pugio* and gobies abundance and biomass, which were the only taxa observed to exhibit post-spill declines. Although only light oiling was reported in our study marshes, is it possible that multiple stressors acted in concert during 2010 to drive modest declines in some taxa, followed by a subsequent recovery in 2011 when conditions improved.

Livebearers were the only taxon to exhibit significant differences in abundance and biomass among reef treatments, but both metrics were higher in control plots relative to reef plots and, thus, contrary to our initial predictions. The lack of reef effects for all metrics suggests that the reefs did not enhance nekton communities in adjacent salt-marsh habitats and did not play a role in shielding the shoreline from oil impacts. Despite the lack of reef effects, prior studies in the region have shown that artificial reefs can enhance animal communities in the immediate vicinity of the reefs, and significantly reduce rates of shoreline erosion [23]. The mitigation of shoreline loss ultimately results in the enhancement of marsh habitat area, quality, and the nekton communities that rely on them as foraging grounds, refuge and nursery habitat.

Our study is consistent with prior accounts of little environmental impact of the DWH spill [18], but it contrasts with other reports that show substantial damage followed by variable rates of recovery [24], [7]. These seemingly discordant results suggest that the impacts of massive environmental accidents such as oil spills can be diverse, ranging from inconsequential to large, and depend on the fate, processing and cleaning up of dispersed oil, tolerance thresholds of organisms affected, and idiosyncrasies of exposed habitats (i.e., sub-tidal vs. intertidal, dominant sediment type, community diversity). A large body of data collected following the DWH spill is currently held by the National Resource Damage Assessment (NRDA) process pending further legal action. The release of this data will not only allow us to integrate our data with other regional studies, but will undoubtedly provide a synthesis of what has or has not been affected in the Northern Gulf of Mexico. When put in context with oil spill studies elsewhere, such a synthesis should improve our ability to avert and manage the negative consequences of such accidents. It seems that, in view of the several studies on the DWH oil spill already completed and the many others currently in the works, such a synthesis will occur in due time.

## Materials and Methods

### Study Site, Oyster Breakwaters and Sampling Design

We sampled marsh-associated nekton within a fringing salt-marsh on the northeast shore of Point-aux-Pins, AL (Figure 1). A continuous, fringing band of smooth cordgrass (*Spartina alterniflora*) was present along most of the shoreline and, given the narrow tidal range of the area, constituted the primary habitat of the low intertidal. The adjacent, subtidal mudflat consisted of a patchwork of bare mud, wigeon grass (*Ruppia martima*), and shoalgrass (*Halodule wrightii*).

Four experimental blocks, each containing one reef and one control plot, were established along 1.2-km of marsh-dominated shoreline. The placement of reef and control plots were randomized within each block, and reefs were constructed of loose oyster shell and reinforcing metal fence. Each reef was composed of three, 25-m long reef modules to produce a structure enveloping 65 m of shoreline. Reef and control plots were separated by 75 m and blocks were separated by a minimum of 100 m. Reefs were located 111 m offshore so that the height of the reef crest coincided with mean low water to facilitate recruitment and growth of oysters.

### Nekton Sampling

Tidal fyke nets were used to sample marsh-associated nekton during flood tides. Each net was constructed of seine netting with 0.64-cm mesh, and enveloped 6 m of fringing marsh and stood 0.75 m tall. The back edge of the net extended 3.25 m seaward from the vegetation edge and all organisms were trapped into a 1 m×1 m seine bag staked to the adjacent flat. The traps were designed to permit lateral movement of organisms within the marsh vegetation as the marsh surface flooded and to capture all organisms as they exit the marsh vegetation on the subsequent falling tide (similar to those used by [25]). One net was randomly positioned at the edge of the vegetation within each plot (i.e., one net per control and reef plot; *n* = 8 nets per sampling period) during low water and left in place for 24 hr. The counts obtained in the traps are indicative of the abundance of organisms that utilized 6 m of linear marsh fringe during a single tidal cycle.

All organisms collected from the traps were immediately placed on ice and transported to the laboratory where they were identified to species, measured and enumerated. Biomass estimates were obtained by pooling species within traps and drying the samples at 70°C for 24–48 hr (measured to the nearest 0.001 gram). This study was carried out in accordance with the recommendations in the Guide for the care and Use of Laboratory Animals of the National Institutes of Health. All necessary permits were obtained for the described field studies by the Alabama Department of Conservation and Natural Resources and our sampling protocol was approved by the United States Army Corp of Engineers (Permit Number SAM-2009-00203-JEB).

Samples were collected once every one to three months for three years. Most of the studied organisms recruit to the marsh from the open waters of the Gulf, or return to the intertidal zone from deeper estuarine habitats, during the early spring months (i.e., March or April; [26], [27]. Therefore, we defined annual periods as follows: pre-spill (year 1): August 2009–February 2010; post-spill 1 (year 2): February 2010–February 2011; post-spill year 2 (year 3): February 2011–February 2012. Although the DWH spill did not occur until April 20, 2010, the number of organisms in the marsh was minimal at the time of the accident and did not peak until mid- to late summer 2010 when impacts on recruitment would become evident. It is important to note that our pre-spill dataset began in August 2009, a period of peak nekton recruitment, and the lack of early, low-abundance months provided for a conservative test between pre- and post-spill years.

### Statistical Analysis

All organisms were categorized as transient or resident taxa based on their use of habitats outside of the estuary and, thus, by their potential risk of exposure to oiling. We compared abundance and biomass of individual transient and resident species, as well as communities, using a partly nested, mixed model Analysis of Variance (ANOVA) among years and between reef treatments. Sampling date was nested within year to account for within-year (i.e., seasonal) variability. Year and reef treatment were defined as fixed factors and sampling date and block were defined as random factors. Separate analyses were conducted for transient and resident organisms. Data transformations were applied as necessary to meet the assumptions of parametric statistics.

We focused our analysis on numerically dominant taxa. Catches of the resident grass shrimp, *Palaemonetes pugio*, accounted for 84.9% of our total catch and were highly variable among sampling days. Seven species or groups of species contributed >1% to the total catch (residents and transients) when *P. pugio* was removed from the data set (Table S1 in File S1). Separate abundance and biomass analyses were conducted for the following transient species (percent contributions to total catch after the removal of *P. pugio*): *Callinectes sapidus* (45.15%), penaeid shrimp (36.04%), and the blackcheeck tonguefish, *Symphurus plagiusa* (1.16%); and for the following groups of residents: *Palaemonetes pugio*, livebearers (7.63%) and gobies (4.56%). Livebearers, which included members of the families fundulidae, poeciliidae and cyprinodontidae, were pooled to assess potential declines in abundance resulting from reproductive and genetic impacts, which have been reported by Whitehead et al. [28] in coastal Louisiana. Gobies included members of the family gobiidae, which utilize marsh structure as a primary habitat. White mullet, *Mugil curema*, constituted 4.85% of the total catch, but were not considered for among-year comparisons since 79.9% of all specimens caught were from a single trap in August 2009.

Total measures of community abundance and biomass (including *P. pugio*) were compared among years and between reef treatments. We then compared the abundance and biomass of transient and resident species with grass shrimp removed from the catch data to discern variability in habitat use by other species that might otherwise have been masked by the disproportionate contribution made by *P. pugio*. Separate analyses were performed for transient and resident communities.

Whenever we did not detect significant differences among years using our general model detailed above, we used a one-way ANOVA to compare maximum abundance values and biomass among years to ascertain whether peak abundance and biomass differed significantly among years despite not finding significant differences in the yearly means. Pair-wise comparisons among years were performed when pertinent.

Lastly, we compared nekton communities among years and reef treatments using a 2-way Analysis of Similarity (ANOSIM) on a fourth-root transformed Bray-Curtis similarity matrix of species abundance and biomass. Multidimensional scaling (MDS) ordination was used to visualize differences among factor levels using PRIMER v6.1.5.

## Supporting Information

File S1 **Supporting Tables S1–S7.**
(DOCX)
