# Supplementary material for: Interannual Recruitment Dynamics for Resident and Transient Marsh Species: Evidence for a Lack of Impact by the Macondo Oil Spill
Source: PLoS One. 2013 Mar 13;8(3):e58376. doi: 10.1371/journal.pone.0058376 (PMC3596379; doi:10.1371/journal.pone.0058376)
Supplement: File S1 — Supporting Tables S1–S7. (DOCX) [file pone.0058376.s001.docx]

**Table S1** Relative frequencies and resident/transient classifications of invertebrates and fishes collected from fringing salt-marsh habitat in coastal Alabama from 2009‑2011.

| **Category** | **Scientific Name** | **% Freq** | **References** |
| --- | --- | --- | --- |
| Resident Species | *Palaemonetes pugio* | 97.55 | [1] |
|  | *Fundulus grandis* | 30.81 | [2] |
|  | *Ctenogobius boleosoma* | 20.61 | [3] |
|  | *Fundulus majalis* | 12.76 | [2] |
|  | *Gobiosoma bosc* | 9.91 | [4] |
|  | *Cyprinodon variegatus* | 7.07 | [5] |
|  | *Gobiesox strumosus* | 3.93 | [6] |
|  | *Cynoscion nebulosus* | 3.34 | [7] |
|  | *Bairdiella chrysoura* | 2.55 | [8] |
|  | *Menidia beryllina* | 1.86 | [9] |
|  | *Poecilia latipinna* | 1.28 | [10] |
|  | *Adinia xenica* | 0.98 | [11] |
|  | *Fundulus similis* | 0.88 | [2] |
|  | *Syngnathus spp.* | 0.88 | [12] |
|  | *Bathygobius soporator* | 0.79 | [13] |
|  | *Cynoscion arenarius* | 0.79 | [14] |
|  | *Fundulus confluentus* | 0.39 | [2] |
|  | *Fundulus pulverous* | 0.39 | [2] |
|  | *Lucania parva* | 0.20 | [15] |
|  | *Fundulus jenkinski* | 0.10 | [2] |
|  | *Gambusia affinis* | 0.10 | [2] |
|  | *Heterandria formosa* | 0.10 | [2] |
|  | *Opsanus beta* | 0.10 | [16] |
|  | *Sphaeroides parva* | 0.10 | [17] |
|  | *Synodus* sp*.* | 0.10 | [18] |
|  |  |  |  |
| Transient Species | *Callinectes sapidus* | 50.46 | [1] |
|  | *Penaeid Shrimp* | 39.59 | [1] |
|  | *Mugil curema* | 5.65 | [19] |
|  | *Symphurus plagiusa* | 1.33 | [20] |
|  | *Leiostomus xanthurus* | 0.99 | [21] |
|  | *Menticirrhus americanus* | 0.83 | [8] |
|  | *Anchoa mitchilli* | 0.52 | [22] |
|  | *Lutjanus griseus* | 0.15 | [23] |
|  | *Lagodon rhomboides* | 0.13 | [24] |
|  | *Paralichthys lethostigma* | 0.10 | [25] |
|  | *Mugil cephalis* | 0.08 | [19] |
|  | *Citharichthys spilopterus* | 0.05 | [26] |
|  | *Menticirrhus saxatalis* | 0.03 | [27] |
|  | *Ophidion* sp*.* | 0.03 | [28] |
|  | *Prionotus* sp*.* | 0.03 | [29] |
|  | *Micropogonias undulates* | 0.02 | [30] |
|  | *Paralichthys albigutta* | 0.02 | [25] |

Footnotes: Resident and transient classifications based on habitat use by spawning adults, eggs and/or larvae. *P. pugio* constituted 97.55% of the total resident organisms caught (*N*=41,510); frequencies of remaining residents are based on total resident abundance with *P. pugio* removed (*n*=1019). Frequencies for transient organisms are based on the total number of transient organisms sampled (*n*=6161).

Literature Cited for Table S1:

1. Peterson MS, Comyns BH, Hendon JR, Bond PJ, Duff GA (2000) Habitat use by early life-history stages of fishes and crustaceans along a changing estuarine landscape: Differences between natural and altered shoreline sites. Wetl Ecol Manag 8: 209-219.

2. McEachran JD, Fechhelm JD (1998) Poeciliidae: Livebearers. In: Fishes of the Gulf of Mexico, Volume 1: Myxiniformes to Gasterosteiformes. Austin, TX: University of Texas Press. pp. 926.

3. Carle KJ, Hastins PA (1982) Selection of meiofaunal prey by the darter goby, *Gobionellus boleosoma* (Gobiidae). Estuaries 5: 316‑318.

4. Hendon JR, Peterson MS, Comyns BH (2000) Spatio-temporal distribution of larval *Gobiosoma bosc* in waters adjacent to natural and altered marsh-edge habitats of Mississippi coastal waters. B Mar Sci 66: 143-156.

5. Nicoletto PF, Linscomb SH (2008) Sound production by the sheepshead minnow, *Cyprinodon variegatus*. Environ Biol Fish 81: 15-20*.*

6. Allen LG (1984) Gobiesociformes: Development and relationships. In: Moser HG, Richards WJ, Cohen DM Fahay MP, Kendall, Jr. AW, editors. Ontogeny and systematics of fishes. Spec. Publ. 1, Am. Soc. Ichthyol. Herpetol. Kansas: Allen Press. pp. 629-636.

7. Wenner CA, Archambault J (1996) Spotted seatrout: Natural history and fishing techniques in South Carolina. (Educational Report No. 18). Marine Resources Research Institute: South Carolina Department of Natural Resources.

8. Johnson GD (1978) Development of fishes of the Mid-Atlantic Bight: An atlas of egg, larval and juvenile stages*.* In: Volume IV: Carrangidae through ephippidae. Office of Biological Services FWS/OBS-78/12. Ft. Collins, Colorado: US Fish and Wildlife Service.

9. Hubbs C (1982) Life history dynamics of *Menidia beryllina* from Lake Texoma. Am Midl Nat 107: 1-12.

10. Felley JD, Daniels GL (1992) Life history of the sailfin molly (*Poecilia latipinna*) in two degraded waterways of southwestern Louisiana. Southwest Nat 37: 16‑21.

11. Hastings RW, Yerger RW (1971) Ecology and life history of the diamond killifish, *Adenia xenica* (Jordan and Gilbert). Am. Mid. Nat. 86: 276‑291.

12. Sweat LH (2009). Species Inventory: *Sygnathus louisianae*. Smithsonian Marine Station: Available: <http://www.sms.si.edu/irlspec/Syngna_louisi.htm> Accessed April 13, 2012.

13. Tavolga WN (1956) Pre-spawning behavior in the gobiid fish, Bathygobius soporator. Behaviour 9: 53-74.

14. Robins CR, Ray GC (1986) A field guide to Atlantic coast fishes of North America. Boston: Houghton Mifflin Company. 354 p.

15. Jordan F (2002) Federation field and laboratory evaluation of habitat use by rainwater killifish (*Lucania parva*) in the St. Johns River Estuary, Florida. Estuaries 25: 288-295.

16. Barimo JF, Scrafy JE, Frezza PE (2007) Habitat use, urea production and spawning in the gulf toadfish, *Opsanus beta*. Mar Biol 150: 497‑508.

17. Chakrabarty P, Lam C, Hardman J, Aaronson J, House PH, Janies DA (2012) SpeciesMap: a web-based application for visualizing the overlap of distributions and pollution events, with a list of fishes put at risk by the 2010 Gulf of Mexico oil spill. Biodivers Conserv 21: 1865-1876.

18. Cruz-Escalona, V. H. (12/01/2005). "Feeding habits and trophic morphology of inshore lizardfish (Synodus foetens) on the central continental shelf off Veracruz, Gulf of Mexico". Journal of applied ichthyology (0175-8659), 21 (6), p. 525.

19. Ibáñez Aguirre AL, Gallardo-Cabello M (2004) Reproduction of *Mugil cephalus* and *M. curema* (pisces: Mugilidae) from a coastal lagoon in the Gulf of Mexico. B Mar Sci 75: 37-49.

20. Switzer TS (2003) The ecology of two estuarine-dependent tonguefishes, the blackcheek tonguefish (Symphurus plagiusa) and the offshore tonguefish (*S. civitatium*), in coastal Louisiana*.* (Unpublished Doctor of Philosophy). Louisiana State University, Ann Harbor, MI. (3135643)

21. Currin BM, Reed JP, Miller JM (1984) Growth, production, food consumption, and mortality of juvenile spot and croaker: A comparison of tidal and nontidal nursery areas. Estuaries 7: 451‑459.

22. Linquist DC, Shaw RF, Hernandez, Jr. FJ (2005) Distribution patterns of larval and juvenile fishes at offshore petroleum platforms in the north-central Gulf of Mexico. Estuar Coast Mar Sci 62: 655‑665.

23. Guevara E, Álvarez H, Mascaró M, Rosas C, Sánchez A (2007) Hábitos alimenticios y ecología trófica del pez *Lutjanus griseus* (pisces: Lutjanidae) asociado a la vegetación sumergida enla laguna de términos, Campeche, México. Rev Biol Trop 55: 989-1004.

24. Nelson, GA (2002) Age, growth, mortality, and distribution of pinfish (L*agodon rhomboides*) in Tampa Bay and adjacent Gulf of Mexico waters. Fish B-NOAA *,* 100: 582-592.

25. Packer DB, Griesbach SJ, Berrien PL, Zetlin CA, Johnson DL, Morse WW (1999) Essential fish habitat source document: Summer flounder, *Paralichthy dentatus*, life history and habitat characteristics*.* (Technical Memorandum No. NMFS-NE-151). New Jersey: Northeast fisheries Science Center's Ecosystems processes Division.

26. Castillo-Rivera M, Kobelkowsky A, Chavez, AM (2000) Feeding biology of the flatfish *Citharichthys spilopterus* (Bothidae) in a tropical estuary of Mexico. J Appl Ichthyol 16: 73‑78.

27. Miller MJ, Rowe P.M., Able KW, Schaefer SA (2002) Occurrence and growth rates of young-of-the-year northern kingfish, *Menticirrhus saxatilis*, on ocean and estuarine beaches in southern New Jersey. Copeia 2002: 815‑823.

28. Woodland RJ (2011) Comparing the nursery role of inner continental shelf and estuarine habitats for temperate marine fishes. Estuar Coast Shelf S 99: 61‑73.

29. Jaureguizar AJ (2007) Spatio-temporal distribution of Atlantic searobins (*Prionotus* spp.) in relation to estuarine dynamics (Río de la Plata, Southwestern Atlantic Coastal System)". Estuar Coast Shelf 73: 30

30. McErlean AJ, O'Connor SG, Milhursky JA, Gibson CI (1973) Abundance, diversity and seasonal patterns of estuarine fish populations. Estuar Coast Mar Sci 1: 19-36.

**Table S2**

Title: Results of partly nested, mixed-model ANOVAs testing for differences in nekton abundances among years and between reef treatments for the following resident species: (A) *Palaemonotes pugio*; (B) Livebearers; and (C) Gobies.

Footnote: "Livebearers" includes pooled abundances of: *Adinia xenica*; *Fundulus confluentus*; *F. grandis*; *F. jenkenski*; *F. majalis*; *F. pulvereus*; *Gambusia affinis*; *Heterandria formosa*; *Lucania parva*; and *Cyprinodon variegatus*. "Gobies" includes pooled abundances of: *Bathygobius soporator*; *Ctenogobius boleosoma*; *Evorthodus lyricus*; and *Gobiosoma bosc.*

|  | Source of variation | df | MS | *F* | *P* value |
| --- | --- | --- | --- | --- | --- |
| A | Year | 2 | 11.7712 | 5.57 | 0.016 |
|  | Date(Year) | 15 | 2.1134 | 6.36 | <0.001 |
|  | Reef | 1 | 0.0155 | 0.05 | 0.830 |
|  | Year x Reef | 2 | 1.1399 | 3.43 | 0.036 |
|  | Block | 3 | 1.2316 | 3.70 | 0.014 |
|  | Error | 120 | 0.3324 |  |  |
|  |  |  |  |  |  |
| B | Year | 1 | 0.1843 | 0.38 | 0.691 |
|  | Date(Year) | 15 | 0.4871 | 3.42 | <0.001 |
|  | Reef | 1 | 1.0149 | 7.12 | 0.009 |
|  | Year x Reef | 2 | 0.2391 | 1.68 | 0.191 |
|  | Block | 3 | 0.2382 | 1.67 | 0.177 |
|  | Error | 120 | 0.1426 |  |  |
|  |  |  |  |  |  |
| C | Year | 2 | 1.28612 | 4.13 | 0.037 |
|  | Date(Year) | 15 | 0.3111 | 3.72 | <0.001 |
|  | Reef | 1 | 0.0141 | 0.17 | 0.682 |
|  | Year x Reef | 2 | 0.0719 | 0.86 | 0.426 |
|  | Block | 3 | 0.09015 | 1.08 | 0.361 |
|  | Error | 120 | 0.0836 |  |  |

**Table S3**

Title: Results of partly nested, mixed-model ANOVAs testing for differences in nekton abundances among years and between reef treatments for the following transient species: (A) *Callinectes sapidus*; (B) penaeid shrimp; and (C) *Symphurus plagiusa*.

Footnote: "Penaeid shrimp" includes pooled abundances of *Litopenaeus setiferus* and *Farfantepenaeus aztecus*.

|  | Source of variation | df | MS | *F* | *P* value |
| --- | --- | --- | --- | --- | --- |
| A | Year | 2 | 4.6463 | 3.69 | 0.050 |
|  | Date(Year) | 15 | 1.2597 | 10.73 | <0.001 |
|  | Reef | 1 | 0.2599 | 2.21 | 0.139 |
|  | Year x Reef | 2 | 0.1235 | 1.05 | 0.352 |
|  | Block | 3 | 0.6626 | 5.65 | 0.001 |
|  | Error | 120 | 0.1174 |  |  |
|  |  |  |  |  |  |
| B | Year | 2 | 2.41449 | 2.11 | 0.156 |
|  | Date(Year) | 15 | 1.14572 | 14.79 | <0.001 |
|  | Reef | 1 | 0.0013 | 0.02 | 0.896 |
|  | Year x Reef | 2 | 0.10959 | 1.41 | 0.247 |
|  | Block | 3 | 0.1132 | 1.46 | 0.229 |
|  | Error | 120 | 0.7748 |  |  |
|  |  |  |  |  |  |
| C | Year | 2 | 0.3600 | 1.86 | 0.190 |
|  | Date(Year) | 15 | 0.1935 | 7.01 | <0.001 |
|  | Reef | 1 | 0.0089 | 0.03 | 0.858 |
|  | Year x Reef | 2 | 0.0041 | 0.15 | 0.863 |
|  | Block | 3 | 0.0801 | 2.90 | 0.038 |
|  | Error | 120 | 0.0276 |  |  |

**Table S4**

Title: Results of partly nested, mixed-model ANOVAs testing for differences in nekton abundances among years and between reef treatments for: (A) total nekton; (B) resident species with *Palaemonetes pugio* removed; and (C) transient species.

|  | Source of variation | df | MS | *F* | *P* value |
| --- | --- | --- | --- | --- | --- |
| A | Year | 2 | 10.4046 | 5.12 | 0.020 |
|  | Date(Year) | 15 | 2.0308 | 9.47 | <0.001 |
|  | Reef | 1 | 0.1406 | 0.66 | 0.420 |
|  | Year x Reef | 2 | 0.5459 | 2.55 | 0.083 |
|  | Block | 3 | 1.0530 | 4.91 | 0.003 |
|  | Error | 120 | 0.2144 |  |  |
|  |  |  |  |  |  |
| B | Year | 2 | 1.6154 | 3.29 | 0.065 |
|  | Date(Year) | 15 | 0.4911 | 3.42 | <0.001 |
|  | Reef | 1 | 0.3776 | 2.63 | 0.107 |
|  | Year x Reef | 2 | 0.2354 | 1.64 | 0.198 |
|  | Block | 3 | 0.4243 | 2.96 | 0.035 |
|  | Error | 120 | 0.1435 |  |  |
|  |  |  |  |  |  |
| C | Year | 2 | 6.0834 | 3.42 | 0.060 |
|  | Date(Year) | 15 | 1.7812 | 14.37 | <0.001 |
|  | Reef | 1 | 0.4552 | 3.67 | 0.058 |
|  | Year x Reef | 2 | 0.1728 | 1.39 | 0.252 |
|  | Block | 3 | 0.7440 | 6.00 | 0.001 |
|  | Error | 120 | 0.1240 |  |  |

**Table S5**

Title: Results of partly nested, mixed-model ANOVAs testing for differences in nekton biomass among years and between reef treatments for the following resident species: (A) *Palaemonotes pugio*; (B) Livebearers; and (C) Gobies.

Footnote: "Livebearers" includes pooled abundances of: *Adinia xenica*; *Fundulus confluentus*; *F. grandis*; *F. jenkenski*; *F. majalis*; *F. pulvereus*; *Gambusia affinis*; *Heterandria formosa*; *Lucania parva*; and *Cyprinodon variegatus*. "Gobies" includes pooled abundances of: *Bathygobius soporator*; *Ctenogobius boleosoma*; *Evorthodus lyricus*; and *Gobiosoma bosc.*

|  | Source of variation | df | MS | *F* | *P* value |
| --- | --- | --- | --- | --- | --- |
| A | Year | 2 | 1397.19 | 4.26 | 0.034 |
|  | Date(Year) | 15 | 327.85 | 3.51 | <0.001 |
|  | Reef | 1 | 306.34 | 3.28 | 0.073 |
|  | Year x Reef | 2 | 482.16 | 5.16 | 0.007 |
|  | Block | 3 | 149.91 | 1.61 | 0.192 |
|  | Error | 120 | 93.37 |  |  |
|  |  |  |  |  |  |
| B | Year | 1 | 17.96 | 0.29 | 0.752 |
|  | Date(Year) | 15 | 61.69 | 1.32 | 0.201 |
|  | Reef | 1 | 257.52 | 5.51 | 0.021 |
|  | Year x Reef | 2 | 45.65 | 0.98 | 0.379 |
|  | Block | 3 | 4.62 | 0.10 | 0.960 |
|  | Error | 120 | 46.74 |  |  |
|  |  |  |  |  |  |
| C | Year | 2 | 1.0316 | 4.71 | 0.026 |
|  | Date(Year) | 15 | 0.2192 | 1.36 | 0.180 |
|  | Reef | 1 | 0.0164 | 0.10 | 0.751 |
|  | Year x Reef | 2 | 0.0223 | 0.14 | 0.871 |
|  | Block | 3 | 0.4453 | 2.76 | 0.045 |
|  | Error | 120 | 0.1616 |  |  |

**Table S6**

Title: Results of partly nested, mixed-model ANOVAs testing for differences in nekton biomass among years and between reef treatments for the following transient species: (A) *Callinectes sapidus*; (B) penaeid shrimp; and (C) *Symphurus plagiusa*.

Footnote: "Penaeid shrimp" includes pooled abundances of *Litopenaeus setiferus* and *Farfantepenaeus aztecus*.

|  | Source of variation | df | MS | *F* | *P* value |
| --- | --- | --- | --- | --- | --- |
| A | Year | 2 | 1794318 | 0.32 | 0.732 |
|  | Date(Year) | 15 | 5642887 | 2.21 | 0.009 |
|  | Reef | 1 | 689758 | 0.27 | 0.604 |
|  | Year x Reef | 2 | 3321855 | 1.30 | 0.276 |
|  | Block | 3 | 902855 | 0.35 | 0.786 |
|  | Error | 120 | 2552020 |  |  |
|  |  |  |  |  |  |
| B | Year | 2 | 1.7733 | 2.12 | 0.155 |
|  | Date(Year) | 15 | 0.8379 | 13.69 | <0.001 |
|  | Reef | 1 | 0.0153 | 0.25 | 0.618 |
|  | Year x Reef | 2 | 0.1572 | 2.57 | 0.081 |
|  | Block | 3 | 0.0799 | 1.31 | 0.276 |
|  | Error | 120 | 0.0612 |  |  |
|  |  |  |  |  |  |
| C | Year | 2 | 0.1207 | 0.98 | 0.399 |
|  | Date(Year) | 15 | 0.12364 | 5.82 | <0.001 |
|  | Reef | 1 | 0.0002 | 0.01 | 0.930 |
|  | Year x Reef | 2 | 0.0031 | 0.15 | 0.865 |
|  | Block | 3 | 0.07550 | 3.56 | 0.016 |
|  | Error | 120 | 0.02124 |  |  |

**Table S7**

Title: Results of partly nested, mixed-model ANOVAs testing for differences in nekton biomass between pre- and post- spill periods, and between reef treatments for: (A) total nekton; (B) resident species with *Palaemonetes pugio* removed; and (C) transient species.

|  | Source of variation | df | MS | *F* | *P* value |
| --- | --- | --- | --- | --- | --- |
| A | Year | 2 | 0.2814 | 1.31 | 0.298 |
|  | Date(Year) | 15 | 0.2143 | 7.54 | <0.001 |
|  | Reef | 1 | 0.08103 | 2.85 | 0.094 |
|  | Year x Reef | 2 | 0.0007 | 0.02 | 0.975 |
|  | Block | 3 | 0.06765 | 2.38 | 0.073 |
|  | Error | 120 | 0.0284 |  |  |
|  |  |  |  |  |  |
| B | Year | 2 | 0.4732 | 1.77 | 0.204 |
|  | Date(Year) | 15 | 0.2677 | 3.39 | <0.001 |
|  | Reef | 1 | 0.0100 | 0.13 | 0.723 |
|  | Year x Reef | 2 | 0.12533 | 1.59 | 0.208 |
|  | Block | 3 | 0.3121 | 3.96 | 0.01 |
|  | Error | 120 | 0.0789 |  |  |
|  |  |  |  |  |  |
| C | Year | 2 | 525733135 | 2.09 | 0.159 |
|  | Date(Year) | 15 | 251896573 | 0.95 | 0.507 |
|  | Reef | 1 | 501047400 | 1.90 | 0.171 |
|  | Year x Reef | 2 | 451294744 | 1.71 | 0.185 |
|  | Block | 3 | 245981456 | 0.93 | 0.427 |
|  | Error | 120 | 263842720 |  |  |
